# Acceleration and High-Speed Running Profiles of Women's International and Domestic Football Matches

**DOI:** 10.3389/fspor.2021.604605

**Published:** 2021-03-25

**Authors:** Jesse Griffin, Timothy Newans, Sean Horan, Justin Keogh, Melissa Andreatta, Clare Minahan

**Affiliations:** ^1^School of Allied Health Sciences, Griffith University, Gold Coast, QLD, Australia; ^2^Exercise and Sports Science, Faculty of Health Sciences and Medicine, Bond University, Gold Coast, QLD, Australia; ^3^Sports Performance Research Centre New Zealand, Auckland University of Technology, Auckland, New Zealand; ^4^Brisbane Strikers Football Club, Brisbane, QLD, Australia

**Keywords:** female athlete, soccer, movement patterns, match demands, GPS

## Abstract

Acceleration and deceleration are important given football is an intermittent sport with constant changes in velocity and direction. It is unclear, however, if the accelerations and decelerations performed by players differ between competition levels. The aim of the present study was to compare the acceleration, deceleration, and high-speed running profiles of players during international and domestic matches and to determine if differences were apparent across playing positions (defenders, midfielders, and attackers). GPS data from 21 Australian women's domestic football league matches over 2 seasons (2016–2018) and 15 Australian women's international matches (2017–2018) were collected and analyzed. Movement pattern data was collected using VX Sport and GPSports 10 Hz GPS receivers. Variables analyzed included: total distance, distance covered high-speed running (16–20 km·h^−1^) distance covered sprinting (> 20 km·h^−1^) and time spent accelerating and decelerating in four predetermined bands (1–2 m·s^−2^, 2–3 m·s^−2^, 3–4 m·s^−2^, and > 4 m·s^−2^). Results revealed that players competing in international matches covered significantly greater total distances, greater high-speed running distances and greater sprinting distances as well as spending a greater duration accelerating in band 4 compared to players in domestic competitions (*p* < 0.05). Players competing in international matches spent significantly less duration decelerating in bands 2 and 3, compared to players in domestic competitions. International defenders and midfielders recorded significantly higher total distances and high-speed running distance compared to players in domestic matches. Our findings suggest that preparing players for international-level competition should include progressive exposure to high-speed running and sprinting distances, as well as high magnitude accelerations. Furthermore, the higher running speeds experienced by players during international matches appears to be a result of less time spent decelerating. The optimal deceleration necessary for specific situations appears important and emphasizes the need for specific deceleration training. The increased effort of high-intensity activity that is required for players competing in international matches affects defenders and midfielders to the greatest degree. Gradual exposure to the increased running demands for midfielders and defenders competing in international matches is needed to improve performance and reduce the potential risk of injury.

## Introduction

Evidence from time-motion analysis studies demonstrates that total distance covered during match-play is similar between international and domestic women's football matches (Gabbett and Mulvey, 2008; Andersson et al., 2010; Gabbett et al., 2013). Nonetheless, players competing in international women's football matches achieve higher running velocities (Gabbett and Mulvey, 2008; Andersson et al., 2010; Gabbett et al., 2013) and cover greater distances at high-speed running (13%) and sprinting (14%) intensities compared to players in domestic competitions (Andersson et al., 2010). International matches also require players to perform longer-duration sprint efforts with shorter recovery periods compared to domestic matches (Gabbett et al., 2013). Since these pioneering time-motion analysis studies (Gabbett and Mulvey, 2008; Andersson et al., 2010; Gabbett et al., 2013), no investigation has utilized the Global Navigation Satellite System (GNSS) to compare acceleration and high-speed running profiles of women's international and domestic football matches. Approval for the utilization of GNSS, specifically Global Positioning System (GPS) technology, within football, has allowed for a greater number of match files and data to be collected and analyzed more time-efficiently (Griffin et al., 2020b). Such comparisons between international and domestic competition levels offer important insights into key differences that can be used to inform talent identification programs and training interventions which may ultimately lead to improved athletic development and performance of female football players.

An important locomotive movement observed during football match-play that has received increasing attention in the literature is acceleration (and deceleration). Maximal acceleration and deceleration are considered “high-intensity” efforts as they impose the greatest physiological and mechanical loading demands on players of any running metric (Bloomfield et al., 2007; Osgnach et al., 2010; Dalen et al., 2016). The metabolic cost of acceleration is higher compared to running at a constant velocity, and as the intensity or number of accelerations increase, so too do the metabolic demands of the movement and also of the match (Osgnach et al., 2010). Likewise, maximal decelerations also produce higher mechanical loads (up to 65% higher) compared to other running metrics such as constant velocity running, due to the eccentric nature of the muscle contractions (McHugh et al., 1999; Dalen et al., 2016; Harper and Kiely, 2018). Accelerations and decelerations contribute significantly to the total high-intensity running distances and sprinting distances of women's football matches and occur more frequently during a match than any other running metric (Mara et al., 2017a; Ramos et al., 2017; Trewin et al., 2018). During women's domestic football competition players have been reported to perform 420 and 430 acceleration and deceleration efforts, respectively (Mara et al., 2017a). Data was collected using 25 Hz Optical Player Tracking and defined acceleration and deceleration as >2 m·s^−2^ and < −2 m·s^−2^, respectively. In contrast, during international competition, players were reported to perform ~200 accelerations (Meylan et al., 2017; Ramos et al., 2017; Trewin et al., 2018) and 170 decelerations (Ramos et al., 2017), with data collection utilizing 10 Hz GPS with an acceleration criteria including >1 m·s^−2^, >2.3 m·s^−2^, and deceleration < −1 m·s^−2^. Direct comparisons between studies may be inappropriate due to the methodological differences in technology used for data collection and criteria to define an acceleration and deceleration. The methodological differences outlined may explain the discrepancies observed.

The analysis of accelerations and decelerations is a key consideration to player load and performance in women's football, given the physiological and mechanical loading and frequency of movements during a match. Comparing accelerations and decelerations during international and domestic women's football matches will determine if a difference exists and if indeed accelerations and decelerations are a distinguishing factor between competition levels. A review of the physical characteristics of female football players has demonstrated that the ability to accelerate and decelerate as measured during field-testing are differentiating factors between international, domestic and sub-elite players (Griffin et al., 2020a). Whether these differences in physical characteristics are also evident in player movement patterns during women's football matches is unknown, conclusive evidence is yet to exist regarding the acceleration and deceleration of players during international and domestic matches. Therefore, the aim of the present study was to examine acceleration, deceleration and high-speed running profiles of players during women's international and domestic football matches. A secondary aim was to examine the effect of playing position on acceleration, deceleration, and high-speed running profiles of players during international and domestic matches.

## Methods

### Subjects

Fifteen female football players (age: 25.7 ± 3.1 years, height: 167.5 ± 7.7 cm, body mass: 61.3 ± 6.2 kg) from the same club team in the Australian women's domestic football league and eighteen female football players (age: 25.6 ± 3.7 years, height: 166.7 ± 8.4 cm, body mass: 59.7 ± 6.8 kg) from the Australian women's national football team participated in the present study. Players were analyzed based on three playing positions from domestic (defenders: *n* = 7, midfielders: *n* = 5, and attackers: *n* = 3) and international matches (defenders: *n* = 8, midfielders: *n* = 9, and attackers: *n* = 6). Data for goalkeepers were excluded given the unique running profile and technical skills of that position.

Data from twenty-one matches (eighty-five individual player match files) over two seasons (2016-2018) were collected from the domestic competition. International matches included data from a total of fifteen games (ninety-seven individual player match files) that were collected from the 2017 Algarve Cup in Portugal, the 2017 Tournament of Nations in the United States of America, the 2018 Asian Cup in Jordan and international “friendlies” in Australia. Only data where a player completed the full match (i.e., 90-min) was included in the study. This study was approved by the Griffith University Human Ethics Committee and Football Federation Australia.

### Procedures

Domestic competition movement data were collected during match-play using VX Sport technology (VX live log, Visuallex Sport International, Wellington, New Zealand) whereas GPSports technology (SPI HPU, GPSports, Canberra, Australia) was utilized during international competitions. Individual players positional and time data was collected by attaching the GPS receivers (VX Sport or GPSports), sampling at 10 Hz, between the scapulae of each player using manufacturer designed elastic vests. Both GPS technology used in the present study have been reported to have acceptable accuracy and both between- and within-manufacturer reliability for quantifying movement patterns during team sport (Varley et al., 2012; Delaney et al., 2018). To ensure that we could confidently compare data collected from two different manufacturers, we performed an inter-manufacturer comparison of the raw data as recommended by Malone et al. (2017). This procedure has been demonstrated to be a valid method for analyzing data from different GPS manufacturers (Thornton et al., 2019; Johnston et al., 2020). The inter-manufacturer comparison comprised of nine elite team sport athletes simultaneously wearing the two different GPS receivers during a 30-m sprint testing session with varying distances of deceleration at the end, determining the smallest worthwhile change. The smallest worthwhile change was determined by dividing the standard deviation of all trials by 0.3 as outlined by Hopkins (2004). The highest variability has shown to occur within acceleration and deceleration variables, so the smallest worthwhile change between GPS manufacturers has been reported for the acceleration and deceleration variables (Table 1).

**Table 1:** Comparison of player movement patterns between domestic and international women's football.

**Variable**	**Domestic**	**International**	**SWC**
Total distance (m)	8727.5 ± 282.5	9432.5 ± 262.9^***^	
HSR (16-20 km·h^−1^) (m)	608.5 ± 68.8	766.4 ± 64.0^***^	
Sprinting (> 20 km·h^−1^) (m)	306.3 ± 56.3	363.7 ± 53.0^*^	
**Acceleration Duration (s)**			
Band 1 (1 to 2 m·s^−2^)	553.9 ± 26.3	523.7 ± 24.6^*^	5.6
Band 2 (2 to 3 m·s^−2^)	187.8 ± 14.6	164.3 ± 13.7^**^	3.5
Band 3 (3 to 4 m·s^−2^)	71.8 ± 5.7	50.5 ± 5.3^***^	2.3
Band 4 (> 4 m·s^−2^)	31.6 ± 3.8	39.4 ± 3.5^***^	3.5
**Deceleration Duration (s)**			
Band 1 (−1 to −2 m·s^−2^)	529.9 ± 29.7	544.2 ± 27.9	6.5
Band 2 (−2 to −3 m·s^−2^)	180.9 ± 13.6	162.1 ± 12.7^**^	3.8
Band 3 (−3 to −4 m·s^−2^)	73.6 ± 5.3	53.8 ± 4.9^**^	2.3
Band 4 (< −4 m·s^−2^)	39.2 ± 4.0	42.4 ± 3.7	3.3

*All values are presented as mean ± 95% CI*.

* *p < 0.05*,

** *p < 0.01*,

*** *p < 0.001*.

*HSR, High-speed running; SWC, Smallest worthwhile change*.

Approximately 30 min before the start of the pre-match warm-up, all receivers were switched on to ensure sufficient time for connection with satellites. The same receivers were worn by the same player for each match to reduce the potential of any inter-unit variability. All players were familiar with the data collection procedures and had experience with wearing GPS receivers during training sessions and matches. Data collection started on the referee's whistle, to commence each half and only included the first 45 min of each half. Data for injury time was excluded so that the match duration was standardized across all games for both domestic and international matches. After each match, data from the GPS receivers were downloaded using VX Sport software (VX View v5.0.3) and GPSports software (Team AMS, R1_2016_7). To minimize the effect of filtering and data processing differences that occur between the manufacturer's software, the raw data was exported to Microsoft Excel and analyzed using R programming language (Version 3.6.1, Vienna, Austria) (Malone et al., 2017; Thornton et al., 2019). Both the VX Sport and GPSports raw exports were analyzed using the same lines of R script. During data analysis, four acceleration and deceleration zones were created, where band 1 was set between 1 and 2 m·s^−2^, band 2 between 2 and 3 m·s^−2^, band 3 between 3 and 4 m·s^−2^, and band 4 above 4 m·s^−2^ based on previous research (Akenhead et al., 2013; Curtis et al., 2018; Harper et al., 2019). The four deceleration zones were identical to their respective acceleration zones, with the difference being these values were negative e.g., between −1 and −2 m·s^−2^. The present study utilized duration accelerating or decelerating (as opposed to frequency or distance) as the primary outcome measure for accelerations and decelerations. It has been demonstrated that the total cumulative distance covered decelerating may not be a true representation of deceleration, given that a player is aiming to cover less distance while decelerating as opposed to more (Harper and Kiely, 2018; Newans et al., 2019). To be valid, all acceleration and deceleration efforts required a minimum duration of 0.2 s with only one acceleration or deceleration effort permitted within a single 1 s period. Sprint analysis research has demonstrated that peak acceleration occurs within the first 0.2 s immediately after the start of a sprint effort from a static starting position (Di Prampero et al., 2005). Furthermore, a minimum duration of 0.2 s has been used in acceleration based research during team-based sports (Coutts et al., 2015; Buchheit and Simpson, 2017). Analysis of high-speed running and sprinting data was based on the pre-defined cut-offs of 16–20 km·h^−1^ and > 20 km·h^−1^, respectively. These thresholds are in agreement with previous investigations of women's football (Griffin et al., 2020b).

### Statistical Analysis

Statistical analysis involved the use of linear mixed models, with significance set at an alpha level of 0.05. Each GPS metric was set as the outcome variable, competition level, and playing position set as a fixed effect, and the player and match were set as random effects. Linear mixed models were conducted in R programming language using the *lme4* package, while the *afex* package was used to calculate confidence intervals and *p*-values.

## Results

The results of the linear mixed models comparing running metrics of players during domestic vs. international football matches are shown in Table 1. Players in international-level matches covered greater distances for all three locomotive metrics (i.e., total distance, high-speed running, and sprinting) compared to players in domestic matches. For accelerations, players competing in international matches spent less duration in band 1, 2, and 3, but a greater duration in band 4. For decelerations, players in international matches spent less duration in bands 2 and 3, compared to players in domestic matches.

**Figure 1:**
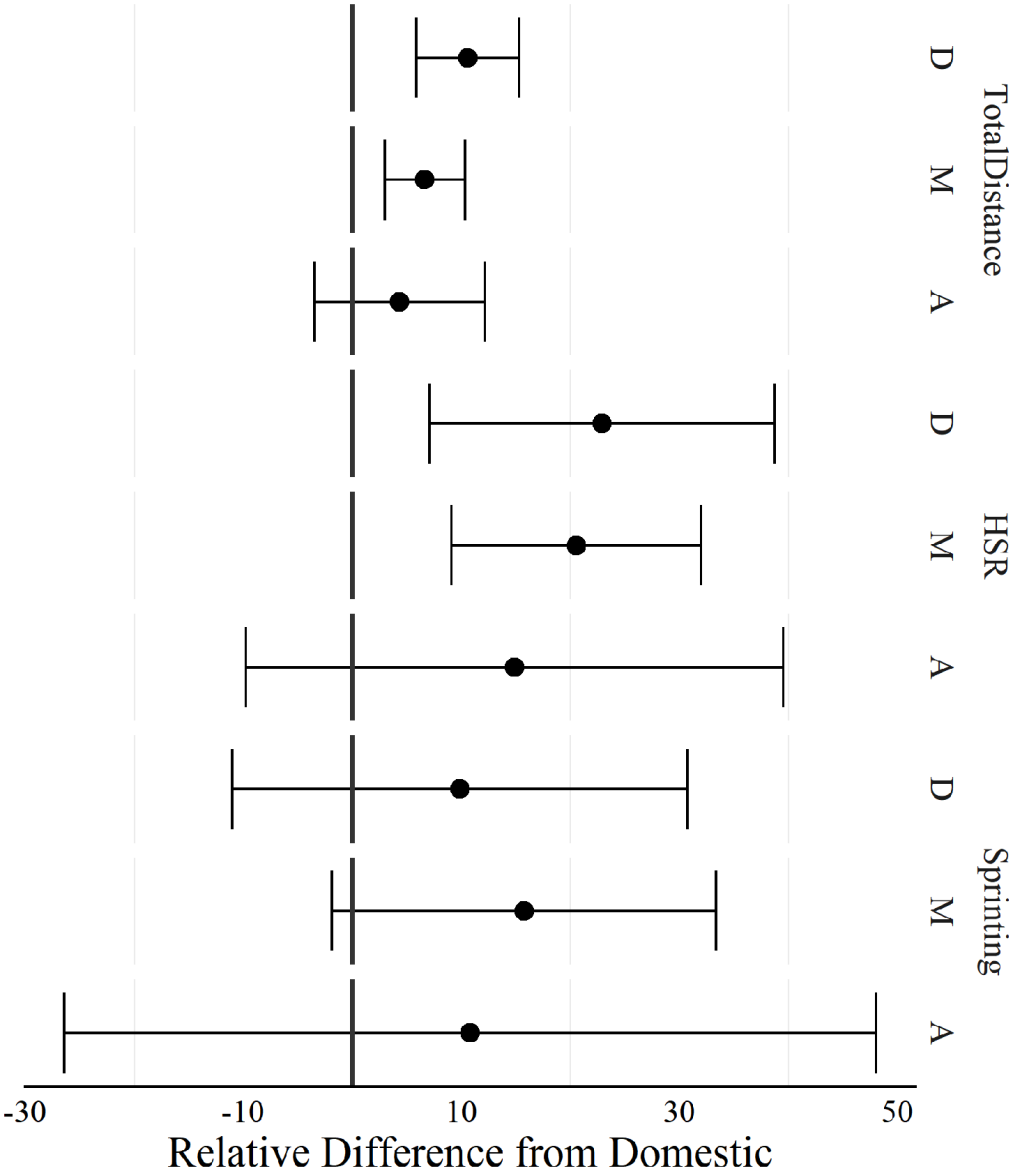
Positional comparison of total distance, high-speed running, and sprinting between players competing in domestic and international women's football matches. HSR, High-speed running (16–20 km·h^−1^); sprinting (>20 km·h^−1^); D, Defenders; M, Midfielders; A, Attackers. A negative difference indicated, domestic was greater than international, a positive difference indicated international was greater than domestic.

The percent difference between competition levels for total distance, high-speed running, and sprinting across playing positions are displayed in Figure 1. Players competing in international matches (defenders and midfielders) recorded higher total distances and high-speed running distance compared to players during domestic competitions.

**Figure 2:**
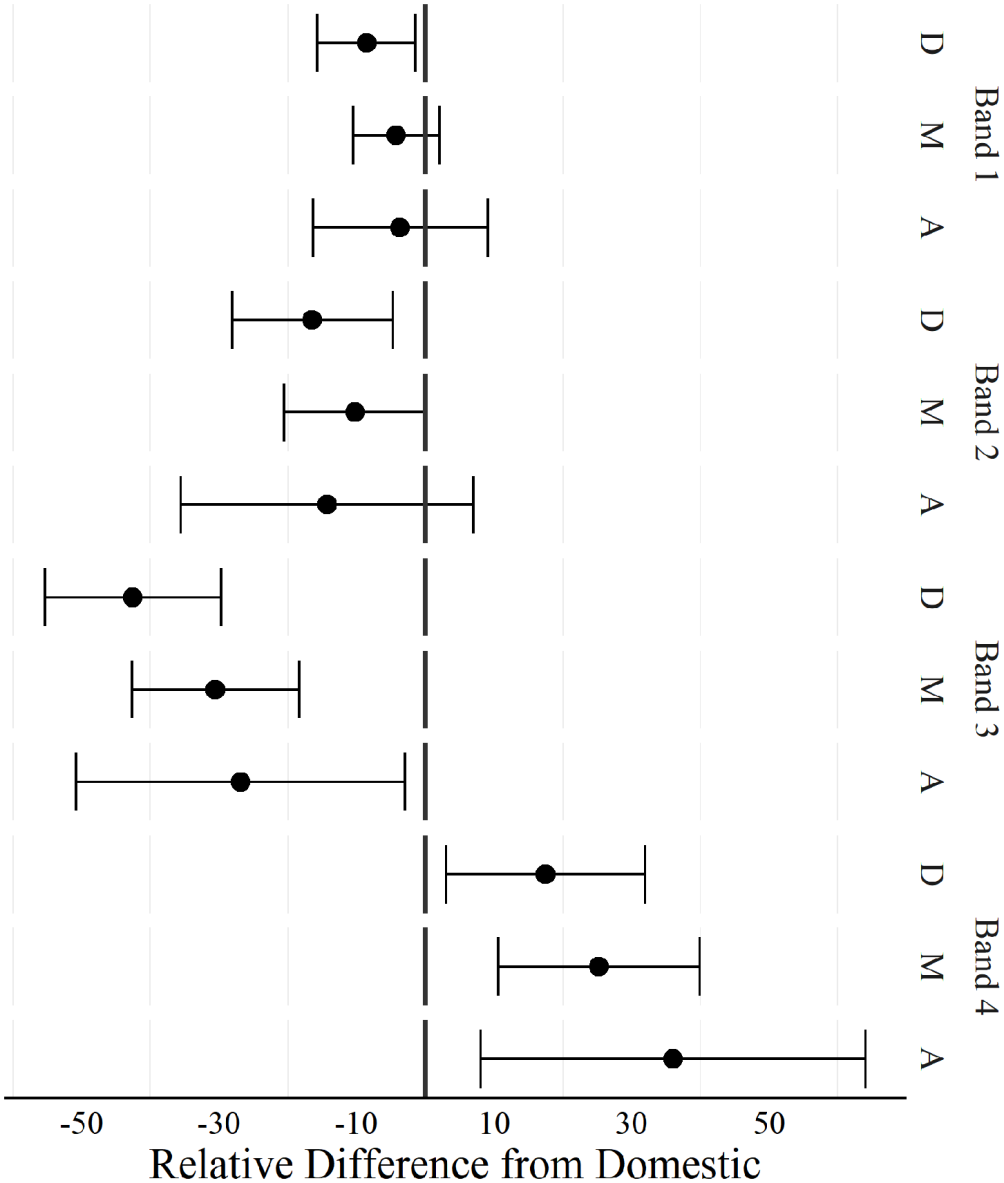
Positional comparison of duration spent accelerating at different intensities between players competing in domestic and international women's football matches. Band 1 (1–2 m·s^−2^), band 2 (2–3 m·s^−2^), band 3 (3–4 m·s^−2^), band 4 (>4 m·s^−2.^). D, Defenders; M, Midfielders; A, Attackers. A negative difference indicated, domestic was greater than international, a positive difference indicated international was greater than domestic.

**Figure 3:**
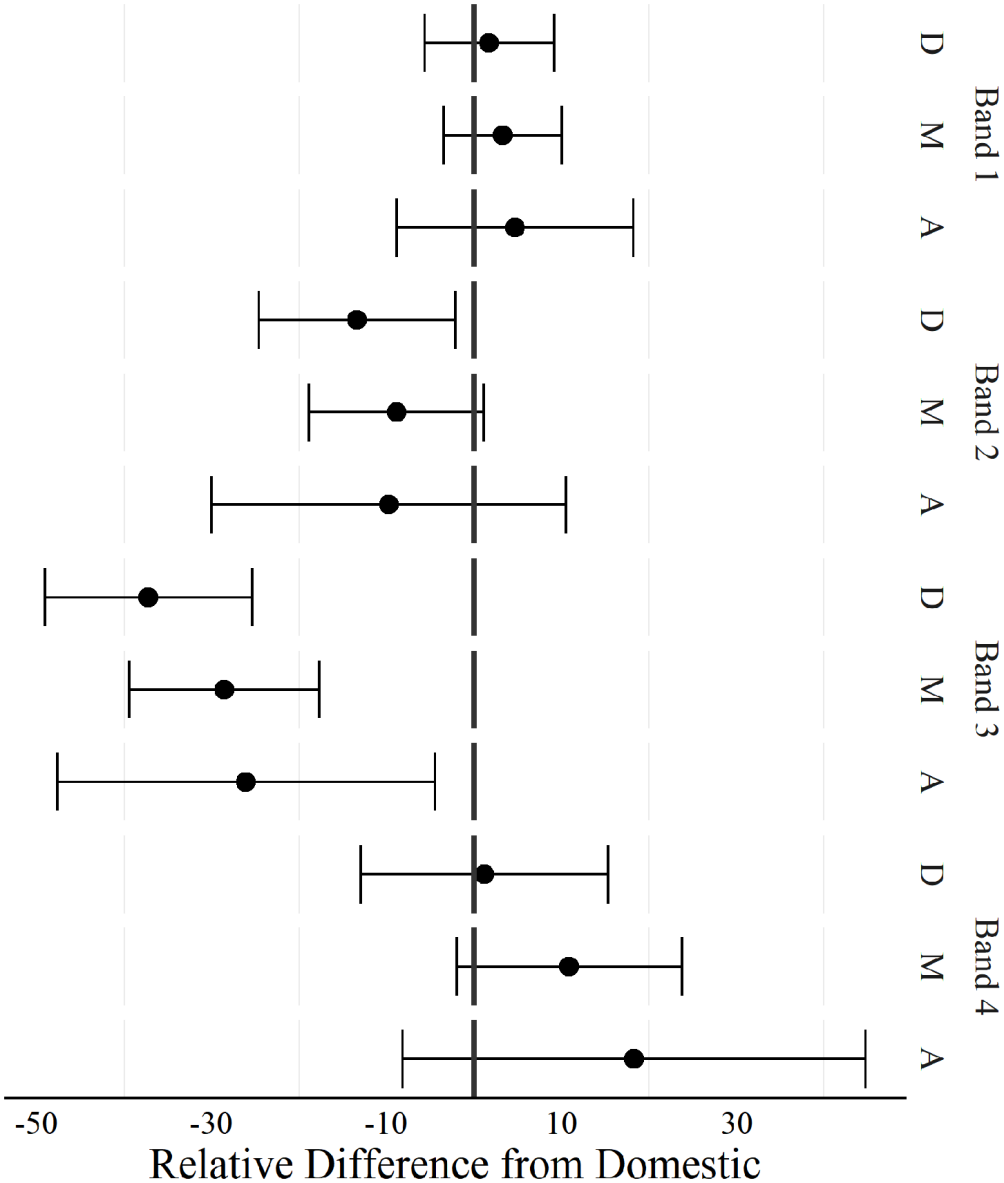
Positional comparison of duration spent decelerating at different intensities between players competing in domestic and international women's football matches. Band 1 (−1 to −2 m·s^−2^), band 2 (−2 to −3 m·s^−2^), band 3 (−3 to −4 m·s^−2^), band 4 (< −4 m·s^−2.^). D, Defenders; M, Midfielders; A, Attackers. A negative difference indicated, domestic was greater than international, a positive difference indicated international was greater than domestic.

The positional differences in acceleration and deceleration between competition level are displayed in Figures 2, 3 respectively. Defenders performed greater duration accelerating in band 1 and 2 during domestic matches compared to international matches. All playing positions during international matches recorded lower durations in acceleration band 3 and spent longer durations accelerating in band 4 compared to domestic matches.

Defenders spent a higher duration in deceleration band 2 during domestic matches compared to international matches. Duration spent decelerating in band 3 was also lower across all positions for international matches.

## Discussion

To the author's knowledge, this is the first study to utilize GPS to make comparisons between international and domestic competition levels and among playing positions as well as to analyze all data outside of the manufactures software to allow for the same analysis and future comparisons to the present study. The present study demonstrated that during international women's football matches, players cover greater distances at faster speeds compared to players in domestic-level competitions. Overall, players competing in international-level matches spent a greater duration accelerating in band 4, covered greater distances at high-speed running and sprinting intensities, and covered greater total distances.

Maximal or near-maximal acceleration is an important precursor to high-speed running and sprinting during football matches, particularly given that the majority of sprinting distance is covered over distances of <10 m (Akenhead et al., 2013; Mara et al., 2017a,b). The present study found that players competing in international matches spent 25% greater duration in acceleration band 4 compared to players competing in domestic competitions. While it could be perceived that an actual difference of ~8 s between players during domestic and international competition might be of no practical significance in isolation, put into context of only 30–40 s currently occurring in the entire match, an increase of 25% is substantial and likely to physiologically impact a player especially in conjunction with additional running demands of a match. Furthermore, the increase between competition levels is for the highest intensity of accelerations and as highlighted in the literature, higher magnitude accelerations impose higher mechanical and physiological load on players (Osgnach et al., 2010, Dalen et al., 2016).

The increased acceleration capacity of players competing in international matches is supported through sprint testing, with players competing in international matches faster over 10 and 20 m compared to players in domestic competitions (Gabbett, 2010; Haugen et al., 2012; Griffin et al., 2020a). The faster 10 and 20 m sprint times of players competing in international matches indicate they have a higher acceleration capacity from a standing start. Further research, is needed to conclusively determine if players competing in international competitions also have a greater acceleration ability when performing accelerations from a “rolling start.” It is clear from our results, that acceleration ability is a key characteristic of female football players competing in international matches, and that coaching and high-performance staff might focus on exposing players to high acceleration activities.

Deceleration is also a critical component of football and is most commonly performed by a player before they undertake a change of direction (COD). Decelerations account for ~15% of total game duration for players competing in both domestic and international matches, suggesting substantial cumulative loads are placed on the lower-body musculoskeletal system during a match and over a season (McHugh et al., 1999). With deceleration ability being a critical mediator of load-related injuries (Harper and Kiely, 2018), it is important that players are adequately prepared for the demands of competition (particularly at an international-level) through the appropriate level of exposure to high deceleration activities before and during the season. An interesting observation from our results was that players competing in international matches spent less duration decelerating in band 2 (12%) and band 3 (32%) compared to players in domestic competitions. The faster intermittent running speeds of players during international matches appears to be a result of less time spent decelerating, which is intuitive given that a high duration of decelerations would cause reductions in speed. The ability to decelerate maximally and come to a stop or reduce velocity faster than your opponent will always be important in team-sport (Hagerman, 2005), it appears though that the ability to decelerate specific to the situation is more important and allows for the preservation of greater speed.

In agreement with previous research (Gabbett and Mulvey, 2008; Andersson et al., 2010), our findings revealed that players competing in international matches performed greater high-speed running and sprinting distances compared to players in domestic competitions. Players competing in international matches performed 26% more distance at high-speed, which is consistent with the 28% difference previously reported for players competing in international matches compared to players in domestic competitions (Mohr et al., 2008). The distance covered at high-speed running and sprinting during international matches presented in the current study was similar to previously reported distances of 755 m of high-speed running and 306 m of sprinting (Ramos et al., 2017). We observed that total distance covered by players was greater during international matches compared to domestic matches. While previous studies have reported no differences in total running distance of players competing in international and domestic women's football matches (Gabbett and Mulvey, 2008; Andersson et al., 2010), a possible explanation may be the differences in technology utilized, with the present study being the first to use GPS to quantify the running profiles of players during domestic and international matches. It is imperative that players are conditioned for the increased demands of international matches. This may require players increasing their aerobic capacity to cope with the higher total distances and increasing their anaerobic capacity to produce the greater high-speed running and sprinting distances.

Positional differences and requirements have been demonstrated to exist in women's football matches (Griffin et al., 2020b). Comparisons between playing positions demonstrate that attacker's movement patterns, except for the duration spent accelerating and decelerating in band 4, were similar or higher during domestic matches. Attackers often perform more high-speed running and sprinting than other positions (DeWitt et al., 2018; Griffin et al., 2020b) however, both of these metrics demonstrate the greatest between-game variability (CV = 33%, 53%, respectively) (Trewin et al., 2018). Our results are also consistent with previous literature, where attackers demonstrated the highest variability between competition levels for all movement patterns. The results found suggest that with increased high intensity accelerations (band 4), attackers may be more prepared for the transition from domestic matches to international matches.

The largest differences in playing positions between competition levels were apparent for defenders and midfielders. When international matches are compared to domestic matches, defenders and midfielders are required to perform more high-speed running, total distance, and greater time spent accelerating in band 4. The increased speed of players competing in international matches, therefore, requires greater movement patterns of defenders and midfielders. Additionally, defenders were the only position to demonstrated greater duration accelerating in band 1 and 2 and decelerating in band 2 during domestic matches compared to international matches. The lower magnitude of accelerations and decelerations highlight the reduced intensity of domestic matches compared to international matches for defenders. The lower intensity of decelerations for defenders may be a direct result of a lack of opportunity to decelerate, given the dependent nature of decelerations on prior velocity and accelerations (Newans et al., 2019). The increase in intensity observed during international matches affects defenders and midfielders to the greatest degree, therefore, it is important if players are to transition to a higher competition level these playing positions specifically are capable of and exposed to the increased intensity required for international matches.

Some inherent limitations warrant acknowledgment in the current study. The collection of GPS data using two different manufacturer GPS receivers is an important limitation, however, collecting data from a domestic and international team meant it was not possible to use receivers from the same manufacturer given each team's contractual obligations and preferences. Despite this, we undertook important steps to ensure standardization of data by implementing measures to minimize the effects of filtering and processing differences between manufacturers, as well as performing an inter-manufacturer comparison to determine the smallest worthwhile change for the variables of interest. The collection of GPS consisted of a small number of subjects, limiting the generalizability of the findings. Despite this limitation, data was based on elite female football players and given the limited number of female players involved in elite football and within a team, this was an unavoidable compromise in order to obtain data applicable to elite female football players.

## Practical Applications

Players competing in international matches should be exposed to a greater volume of high-speed running, sprinting and high magnitude (band 4) accelerations during training to prepare them for the increased demands of an international match. In the current study, players covered 26% more distance high-speed running during international matches compared to domestic matches. This is an important consideration in preparing players for international matches. Specifically, acceleration ability needs to focus on producing higher magnitude (band 4) accelerations, by applying greater force to the ground over a shorter ground contact time. Given acceleration or prior speed are needed for deceleration training, it is recommended that speed and deceleration are trained simultaneously as it may offer the most time-efficient approach to integrating both of these important elements into a time-restricted training program.

## Conclusion

Results from the present study demonstrate that players competing in international matches perform more explosive, faster efforts, with greater outputs of high magnitude (band 4) accelerations, high-speed running, and sprinting. To prepare players for these increased running demands, players need to be progressively exposed to high-intensity activities during training. To maintain the faster speeds that are required during international matches, it appears that the ability to decelerate specific to the situation is important. The use of magnitude bands in the current study provides novel insights into differences between players movement patterns during international and domestic matches, highlighting the need for deeper understanding around the distribution of specific magnitudes of acceleration and deceleration efforts. The increased effort of high-intensity activity that is required for international matches in comparison to domestic matches, affects defenders and midfielders to the greatest degree. Therefore, it is important that these playing positions are gradually exposed to the increased stimulus to improve performance and reduce the potential risk of injury.

## Data Availability Statement

The raw data supporting the conclusions of this article will be made available by the authors, without undue reservation.

## Ethics Statement

The studies involving human participants were reviewed and approved by Griffith University Human Ethics Committee. Written informed consent to participate in this study was provided by the participants' legal guardian/next of kin.

## Author Contributions

JG: project concept, data collection and analysis, and preparation of manuscript. CM: project concept, refining, and synthesizing manuscript. SH: data preparation, refining, and synthesizing manuscript. JK: refining and synthesizing manuscript. TN: data analysis and preparation of results (tables and figures). MA: project concept and provision of data collection. All authors contributed to the article and approved the submitted version.

## Conflict of Interest

The authors declare that the research was conducted in the absence of any commercial or financial relationships that could be construed as a potential conflict of interest.

## References

[B1] Akenhead R. Hayes P. R. Thompson K. G. French D. (2013). Diminutions of acceleration and deceleration output during professional football match play. J. Sci. Med. Sport. 16, 556–561. 10.1016/j.jsams.2012.12.005 23333009

[B2] Andersson H. Randers M. Heiner-Moller A. Krustrup P. Mohr M. (2010). Elite female soccer players perform more high-intensity running when playing in international games compared with domestic league games. J. Strength Cond. Res. 24, 912–919. 10.1519/JSC.0b013e3181d09f21 20300037

[B3] Bloomfield J. Polman R. O'Donoghue P. (2007). Deceleration movements performed during FA Premier League soccer matches. J Sport Sci Med. (Suppl. 10):6. PMC3778701 24149226

[B4] Buchheit M. Simpson B. (2017). Player-tracking technology: half-full or half-empty glass? Int. J. Sports Physiol. Perform. 12(Suppl 2):35–41. 10.1123/ijspp.2016-0499 27967285

[B5] Coutts A. J. Kempton T. Sullivan C. Bilsborough J. Cordy J. Rampinini E. (2015). Metabolic power and energetic costs of professional Australian football match-play. J. Sci. Med. Sport. 18, 219–224. 10.1016/j.jsams.2014.02.003 24589369

[B6] Curtis R. M. Huggins R. A. Looney D. P. West C. A. Fortunati A. Fontaine G. J.. (2018). Match demands of National Collegiate Athletic Association division I men's soccer. J. Strength Cond. Res. 32, 2907–2917. 10.1519/JSC.0000000000002719 29979277

[B7] Dalen T. Ingebrigtsen J. Ettema G. Hjelde G. Wisloff U. (2016). Player load, acceleration, and deceleration during forty-five competitive matches of elite soccer. J. Strength Cond. Res. 30, 351–359. 10.1519/JSC.0000000000001063 26057190

[B8] Delaney J. A. Cummins C. J. Thornton H. R. Duthie G. M. (2018). Importance, reliability, and usefulness of acceleration measures in team sports. J. Strength Cond. Res. 32, 3485–3493. 10.1519/JSC.0000000000001849 28195980

[B9] DeWitt J. Gonzales M. Laughlin M. Amonette W. (2018). External loading is dependent upon game state and varies by position in professional women's soccer. Sci Med Football. 2, 225–230. 10.1080/24733938.2018.1447142

[B10] Di Prampero P. Fusi S. Sepulcri L. Morin J. B. Belli A. Antonutto G. (2005). Sprint running: a new energetic approach. J. Exp. Biol. 208, 2809–2816. 10.1242/jeb.01700 16000549

[B11] Gabbett T. (2010). The development of a test of repeated-sprint ability for elite women's soccer players. J. Strength Cond. Res. 24, 1191–1194. 10.1519/JSC.0b013e3181d1568c 20386127

[B12] Gabbett T. Mulvey M. (2008). Time-motion analysis of small-sided training games and competition in elite women soccer players. J. Strength Cond. Res. 22, 543–552. 10.1519/JSC.0b013e3181635597 18550972

[B13] Gabbett T. Wiig H. Spencer M. (2013). Repeated high-intensity running and sprinting in elite women's soccer competition. Int. J. Sports Physiol. Perform. 8, 130–138. 10.1123/ijspp.8.2.130 22868237

[B14] Griffin J. Horan S. Keogh J. Dodd K. Andreatta M. Minahan C. (2020a). Contextual factors influencing the characteristics of female football players. J Sport Med Phys Fit. 34, 2384–2393. 10.23736/S0022-4707.20.11182-4 32744042

[B15] Griffin J. Larsen B. Horan S. Keogh J. Dodd K. Andreatta M.. (2020b). Women's football: An examination of factors that influence movement patterns. J. Strength Cond. Res. 34, 2384–2393. 10.1519/JSC.0000000000003638 32412968

[B16] Hagerman P. (2005). Putting on the brakes: deceleration training. Strength Cond J. 27, 57–58. 10.1519/00126548-200502000-00011

[B17] Harper D. J. Carling C. Kiely J. (2019). High-intensity acceleration and deceleration demands in elite team sports competitive match play: a systematic review and meta-analysis of observational studies. Sports Med. 49, 1923–1947 10.1007/s40279-019-01170-1 31506901 PMC6851047

[B18] Harper D. J. Kiely J. (2018). Damaging nature of decelerations: do we adequately prepare players? BMJ Open Sport Exerc Med. 4:1. 10.1136/bmjsem-2018-000379 30112183 PMC6089312

[B19] Haugen T. Tonnessen E. Seiler S. (2012). Speed and countermovement-jump characteristics of elite female soccer players, 1995-2010. Int. J. Sports Physiol. Perform. 7, 340–349. 10.1123/ijspp.7.4.340 22645175

[B20] Hopkins W. (2004). How to interpret changes in an athletic performance test. Sportscience. 8, 1–7.

[B21] Johnston R. D. Thornton H. R. Wade J. A. Devlin P. Duthie G. M. (2020). The distribution of match activities relative to the maximal mean intensities in professional rugby league and Australian football. J Strength Cond Res. 10.1519/JSC.0000000000003613. [Epub ahead of print]. 32412969

[B22] Malone J. Lovell R. Varley M. Coutts A. (2017). Unpacking the black box: applications and considerations for using GPS devices in sport. Int. J. Sports Physiol. Perform. 12(Suppl 2):218–226. 10.1123/ijspp.2016-0236 27736244

[B23] Mara J. Thompson K. Pumpa K. Morgan S. (2017a). The acceleration and deceleration profiles of elite female soccer players during competitive matches. J. Sci. Med. Sport. 20, 867–872. 10.1016/j.jsams.2016.12.078 28173971

[B24] Mara J. Thompson K. Pumpa K. Morgan S. (2017b). Quantifying the high-speed running and sprinting profiles of elite female soccer players during competitive matches using an Optical Player Tracking System. J. Strength Cond. Res. 31, 1500–1508. 10.1519/JSC.0000000000001629 28538298

[B25] McHugh M. P. Connolly D. A. J. Eston R. G. Gleim G. W. (1999). Exercise-induced muscle damage and potential mechanisms for the repeated bout effect. Sports Med. 27, 157–170. 10.2165/00007256-199927030-00002 10222539

[B26] Meylan C. Trewin J. McKean K. (2017). Quantifying explosive actions in international women's soccer. Int. J. Sports Physiol. Perform. 12, 310–315. 10.1123/ijspp.2015-0520 27295719

[B27] Mohr M. Krustrup P. Andersson H. Kirkendal D. Bangsbo J. (2008). Match activities of elite women soccer players at different performance levels. J. Strength Cond. Res. 22, 341–349. 10.1519/JSC.0b013e318165fef6 18550946

[B28] Newans T. Bellinger P. Dodd K. Minahan C. (2019). Modelling the acceleration and deceleration profile of elite-level soccer players. Int. J. Sports Med. 40, 331–335. 10.1055/a-0853-7676 30887482

[B29] Osgnach C. Poser S. Bernardini R. Rinaldo R. Di Prampero P. (2010). Energy cost and metabolic power in elite soccer: a new match analysis approach. Med. Sci. Sports Exerc. 42, 170–178. 10.1249/MSS.0b013e3181ae5cfd 20010116

[B30] Ramos G. Nakamura F. Penna E. Coimbra C. (2017). Activity profiles in U17, U20 and senior women's Brazilian national soccer teams during international competitions: are there meaningful differences? J. Strength Cond. Res. 33, 3414–3422. 10.1519/JSC.0000000000002170 28767483

[B31] Thornton H. R. Nelson A. R. Delaney J. A. Serpiello F. R. Duthie G. M. (2019). Interunit reliability and effect of data-processing methods of global positioning systems. Int. J. Sports Physiol. Perform. 14, 432–438. 10.1123/ijspp.2018-0273 30204529

[B32] Trewin J. Meylan C. Varley M. Cronin J. (2018). The match-to-match variation of match-running in elite female soccer. J. Sci. Med. Sport. 21, 196–201. 10.1016/j.jsams.2017.05.009 28595867

[B33] Varley M. Fairweather I. Aughey R. (2012). Validity and reliability of GPS for measuring instantaneous velocity during acceleration, deceleration, and constant motion. J. Sports Sci. 30, 121–127. 10.1080/02640414.2011.627941 22122431

